# Clinical characteristics and COVID-19 outcomes in a regional cohort of pediatric patients with rheumatic diseases

**DOI:** 10.1186/s12969-021-00648-5

**Published:** 2021-11-27

**Authors:** Daniel Clemente, Clara Udaondo, Jaime de Inocencio, Juan Carlos Nieto, Pilar Galán del Río, Antía García Fernández, Jaime Arroyo Palomo, Javier Bachiller-Corral, Juan Carlos Lopez Robledillo, Claudia Millán Longo, Leticia Leon, Lydia Abasolo, Alina Boteanu

**Affiliations:** 1 Pediatric Rheumatology Unit, Hospital Infantil Universitario Niño Jesús, Madrid, Spain; 2 Pediatric Rheumatology Department, Hospital Universitario La Paz, Madrid, Spain; 3 Pediatric Rheumatology Department, Hospital Universitario 12 de Octubre, Madrid, Spain; 4 Rheumatology Department, Hospital Universitario Gregorio Marañon, Madrid, Spain; 5 Rheumatology Department, Hospital Universitario de Fuenlabrada, Madrid, Spain; 6 Rheumatology Department, Hospital Universitario Ramón y Cajal, Madrid, Spain; 7 Rheumatology Department and IDISSC, Hospital Universitario Clinico San Carlos, Martin Lagos s/n, 28040 Madrid, Spain; 8 Health Sciences Faculty, Universidad Camilo Jose Cela, Madrid, Spain

**Keywords:** Childhood-onset rheumatic diseases, COVID-19, Chronic inflammatory arthritis, Autoinflammatory diseases, Multisystemic autoimmune disorders

## Abstract

**Background:**

This study aimed to assess the baseline characteristics and clinical outcomes of coronavirus disease 2019 (COVID-19) in pediatric patients with rheumatic and musculoskeletal diseases (RMD) and identify the risk factors associated with symptomatic or severe disease defined as hospital admission, intensive care admission or death.

**Methods:**

An observational longitudinal study was conducted during the first year of the SARS-CoV-2 pandemic (March 2020–March 2021). All pediatric patients attended at the rheumatology outpatient clinics of six tertiary referral hospitals in Madrid, Spain, with a diagnosis of RMD and COVID-19 were included. Main outcomes were symptomatic disease and hospital admission. The covariates were sociodemographic and clinical characteristics and treatment regimens. We ran a multivariable logistic regression model to assess associated factors for outcomes.

**Results:**

The study population included 77 pediatric patients. Mean age was 11.88 (4.04) years Of these, 30 patients (38.96%) were asymptomatic, 41 (53.25%) had a mild-moderate COVID-19 and 6 patients (7.79%) required hospital admission. The median length of hospital admission was 5 (2–20) days, one patient required intensive care and there were no deaths. Previous comorbidities increased the risk for symptomatic disease and hospital admission. Compared with outpatients, the factor independently associated with hospital admission was previous use of glucocorticoids (OR 3.51; *p* = 0.00). No statistically significant risk factors for symptomatic COVID-19 were found in the final model.

**Conclusion:**

No differences in COVID-19 outcomes according to childhood-onset rheumatic disease types were found. Results suggest that associated comorbidities and treatment with glucocorticoids increase the risk of hospital admission.

## Background

Severe acute respiratory syndrome coronavirus-2 (SARS-CoV-2) infection causes COVID-19 disease, which may present with a myriad of clinical symptoms and signs [1].

Since the first confirmed case of SARS-CoV-2 infection in January 2020, the current COVID-19 outbreak has had considerable impact in Spain, especially in the Madrid region, where a higher incidence of COVID-19 cases has been recorded [2].

The incidence and severity of COVID-19 disease seems to be higher in patients with risk factors such as advanced age and associated comorbidities, mainly hypertension, diabetes, heart disease, and previous respiratory diseases [3–5]. Autoimmune systemic disorders may be considered another risk factor for severity and COVID-19 in these patients has been associated with a higher risk of hospital admission [6].

In contrast with infected adults, most infected children appear to have a milder clinical course, and asymptomatic infections are not uncommon [7]. The admission rate to intensive care for pediatric patients with COVID-19 is around 2–3%, and fatal cases are rare (0,08%) [8, 9].

Children may develop a multisystemic inflammatory syndrome in association with COVID-19 (MIS-C) [10]. However, information on the clinical course of the disease in patients with autoimmune or autoinflammatory conditions is scarce.

Regarding therapies, studies in adult patients with rheumatic and musculoskeletal diseases (RMD) have demonstrated that most synthetic and biological disease-modifying antirheumatic drugs are not associated with worse outcomes in COVID-19. However, patients with poorly controlled active RMD or receiving treatments such as glucocorticoids may have an increased risk of both infection and severe disease [6, 11].

There is growing evidence that pediatric patients with autoimmune/inflammatory diseases receiving immunomodulatory therapy and with SARS-CoV-2 infection are not at a significantly increased risk of poor outcomes [12]. Prior to this study, several small case series of COVID-19 in pediatric patients with RMD have been reported [13, 14]. Evidence on the possible risk factors for poor outcome in pediatric patients with rheumatic disease and COVID-19 and data on outcome of hospital admissions for these patients is scarce.

Therefore, due to a paucity of research data about COVID-19 in pediatric patients with rheumatic diseases, we conducted this study to obtain a general picture of pediatric patients with RMDs and COVID-19 in the region of Madrid. Thus, the purpose was to assess the baseline characteristics and clinical outcomes of COVID-19 in pediatric patients with RMDs and determine the risk factors associated with the development of mild, moderate or severe COVID-19.

## Methods

The COVID-SORCOM registry is a multicenter regional registry established to analyse the clinical characteristics and outcomes of COVID-19 in patients with RMD in Madrid, Spain. Aretrospective observational study of children and adolescents with RMD included in this registry was performed, during the period March 2020–March 2021. Clinical and demographic characteristics, COVID-19 data, and outcomes of these patients were analysed.

The inclusion criteria were age < 18 years, a diagnosis of a pediatric RMD (according to ICD-10) and COVID-19 disease confirmed with a positive SARS-CoV-2 polymerase chain reaction (PCR) test, a positive rapid antigen test or detection of antibodies against SARS-CoV-2. Patients with a clinical diagnosis based only on typical symptoms and epidemiological data during the first weeks of the pandemic due to the lack of widely available diagnostic tests, were also included.

The severity of COVID-19 was defined on the basis of clinical features, laboratory testing, and chest radiograph imaging, distinguishing between asymptomatic infection and symptomatic infection (the latter was further classified as mild, moderate, severe, or critical) [15]. Patients with mild disease were those with symptomatic infection who did not require admission; patients with moderate disease those who required hospital admission due to symptoms, patients with severe disease were those who required intensive care, and patients with critical disease those with severe disease and a poor outcome with significant sequelae or death.

Primary outcomes analysed were presence of symptomatic COVID-19 and hospital admission.

Recorded co-variables included:

**1)** Sociodemographic baseline characteristics including sex, age, and RMD duration.

**2)** Type of RMD, classified into the following 3 groups: a) chronic inflammatory arthritis: juvenile idiopathic arthritis (JIA) (excluding the systemic subtype), enthesitis-related arthritis, and anterior chronic uveitis associated to JIA; b) multisystemic autoimmune disorders: systemic lupus erythematosus, idiopathic inflammatory myopathy, scleroderma and pediatric vasculitis and c) autoinflammatory diseases: systemic JIA, PFAPA syndrome, chronic recurrent multifocal osteomyelitis and monogenic autoinflammatory syndromes (TRAPS, CAPS, FMF, MKD).

**3)** Disease activity according to the attending physician’s criteria (clinical remission on and off medication, low, moderate or high disease activity).

**4)** Baseline comorbidities.

**5)** Treatment for rheumatic disease: a) glucocorticoids (according to dose: 0 mg, 0-10 mg, > 10 mg), b) nonsteroidal anti-inflammatory drugs (NSAIDs), c) conventional synthetic disease-modifying antirheumatic drugs (csDMARDs); d) targeted synthetic/biologic DMARDs (ts/bDMARDs).

For the hospital admission episode for COVID-19, clinical, laboratory, and treatment data until discharge were recorded.

Patient characteristics were expressed as the mean and standard deviation or median and interquartile range for continuous variables; categorical variables were expressed as percentages. Characteristics of patients with asymptomatic COVID-19 and those with symptoms, including need for hospital admission were compared. Continuous variables were analysed using the Mann-Whitney U test, and discrete variables were analyzed using Fisher’s exact test. Univariable logistic regression analyses were performed to assess differences between asymptomatic or symptomatic cases, hospital admissions due to COVID-19 and covariables. Multivariable logistic regression models (adjusted for age, sex) were tested in a stepwise manner to examine the possible effect of sociodemographic, clinical, and therapeutic factors on symptomatic COVID-19 and COVID-19 hospital admissions. The model also included all other variables with a *p* < 0.1 from the univariable regression analysis. The results were expressed as the odds ratio (OR) with 95% confidence intervals. Study data were collected and managed using REDCap electronic data capture tools hosted at SORCOM [16, 17]. The study was approved by the Hospital Ramon y Cajal Institutional Ethics Committee (approval number 136–20).

All analyses were performed using Stata v.13 statistical software (Stata Corp., College Station, TX, USA). A two-tailed *p* value < 0.05 was considered statistically significant.

## Results

### Patient characteristics

In total, 77 pediatric patients with rheumatic diseases and COVID-19 were included in the study.

Of these, 66.23% were diagnosed with a positive SARS-CoV-2 polymerase chain reaction (PCR) test, 27.27% based on detection SARS-CoV-2 antibodies, and 6.49% had a clinical diagnosis. Fifty-five (71.43%) patients were female and the mean age of patients was 11.88 (SD 4.04) years. Mean time since diagnosis of RMD was 5.78 (SD 4.21) years and most of the patients had a diagnosis of oligoarticular or polyarticular JIA with or without associated uveitis (*n* = 42, 54.54%). Other diagnoses were systemic lupus erythematosus (*n* = 8, 10.38%), systemic JIA (*n* = 5, 6.49%) and monogenic autoinflammatory syndrome (*n* = 5, 6.49%).

Additional comorbidities included obesity (2 patients), chronic renal disease (2), asthma (1), chronic heart disease (1), previous macrophage activation syndrome (1) and a duplicated collecting system (1) (Main characteristics of pediatric patients with COVID-19 and rheumatic diseases are shown in Table 1).

**Table 1:** Baseline sociodemographic and clinical characteristics of pediatric patients with COVID-19 and rheumatic diseases

Variable	RMD COVID-19 patients *n* = 77	RMD COVID-19 symptomatic patients *n* = 47	RMD COVID-19 asymptomatic patients *n* = 30	*p* value*
Women, n (%)	55 (71.43)	31 (65.95)	24 (80)	0.20
Age, mean (SD), years	11.88 (4.04)	12.04 (4.22)	11.63 (3.81)	0.53
Clinical remission, n (%)	52 (67.53)	28 (59.57)	24 (80)	0.37
Comorbidities	8 (10.39)	8 (17.02)	0	0.02
Diagnosis, n (%)				0.53
Chronic inflammatory arthritis				
Oligoarticular JIA	21 (27.27)	9 (19.14)	12 (40)	
Oligoarticular JIA with associated uveitis	9 (11.69)	5 (10.63)	4 (13.33)	
Polyarticular JIA	12 (15.58)	8 (17.02)	4 (13.33)	
Enthesitis-related arthritis	4 (5.19)	3 (6.38)	1 (3.33)	
Idiopathic uveitis	2 (2.60)	2 (4.25)	0	
Multisystemic autoimmune disorders				
Systemic lupus erythematosus	8 (10.38)	6 (12.76)	2 (6.66)	
Scleroderma	1 (1.30)	1 (2.12)	0	
Idiopathic inflammatory myopathy	3 (3.90)	2 (4.25)	1 (3.33)	
Pediatric vasculitis	2 (2.60)	1 (2.12)	1 (3.33)	
Autoinflammatory diseases				
Systemic JIA	5 (6.49)	4 (8.51)	1 (3.33)	
Chronic recurrent multifocal osteomyelitis	3 (3.90)	2 (4.25)	1 (3.33)	
PFAPA syndrome	2 (2.60)	2 (4.25)	0	
Monogenic autoinflammatory syndrome	5 (6.49)	2 (4.25)	3 (10)	
Rheumatic disease treatment, n (%)				
NSAIDs, n (%)	3 (3.90)	2 (4.25)	1 (3.33)	0.99
Glucocorticoids, n (%)	9 (11.69)	7 (14.89)	2 (6.66)	0.46
csDMARDs^a^, n (%)	35 (45.45)	22 (46.80)	13 (43.33)	0.81
Ts/bDMARDs^b^, n (%)	40 (51.95)	24 (51.05)	16 (53.33)	0.99

*Abbreviations*: *CI* Confidence Interval, *SD* standard deviation, *JIA* Juvenile idiopathic arthritis, *NSAIDs* Non-steroidal anti-inflammatory drugs, *csDMARD* Conventional synthetic disease-modifying anti rheumatic drugs, *Ts/bDMARDs* target synthetic/biologic disease-modifying anti rheumatic drug; ^a^ Methotrexate (*n* = 26), Hydroxychloroquine (*n* = 7), leflunomide (*n* = 2), Mycophenolate (*n* = 6), Azathioprine (*n* = 1), Cyclophosphamide (*n* = 1), Colchicine (*n* = 1). ^b^Adalimumab (*n* = 11), Tocilizumab (*n* = 4), Canakinumab (*n* = 3), Etanercept (*n* = 15), Infliximab (*n* = 4), Rituximab (*n* = 1), Sarilumab (*n* = 1), Tofacitinib (*n* = 1)

* *p* < 0.05

While 8 of the patients (10.53%) had high RMD activity at the time of COVID-19 diagnosis, most (89.47%) had low activity or were in clinical remission.

Prior to COVID-19 infection, only nine of the patients were taking glucocorticoids (11.68%) and 3 were taking NSAIDs on a regular basis (3.90%). Almost half of the patients were taking csDMARDs, mainly methotrexate (*n* = 26, 33.77%), antimalarial agents (*n* = 7, 9.09%) and mycophenolate mofetil (*n* = 6, 7.79%). Over half of the patients (51.95%) were taking ts/bDMARDs, mainly anti-TNF therapy, etanercept being the most frequently prescribed drug (19.48%), followed by adalimumab (14.29%). Only one patient was taking a Janus-Kinase (JAK) inhibitor. Additionally, 3 patients were taking Angiotensin-converting-enzyme (ACE) inhibitors and two were on oral anticoagulation agents.

### COVID-19 outcomes

Of the 77 patients, 47 had at least one COVID-19-related symptom (mainly fever, headache or cough), while 30 patients were asymptomatic. Symptomatic patients were older (14 [8.5–16] vs 12 [9.5–15]) than asymptomatic ones, without significant differences. No patients were diagnosed with the pediatric multisystemic inflammatory syndrome. Overall, 81.52% of patients maintained all their immunosuppressive treatment without changes. Around 20% of patients went to the emergency room due to their symptoms.

Patients with multisystemic autoimmune disorders were more frequently symptomatic due to COVID-19 (71.4% with symptoms), followed by those with autoinflammatory diseases (66.6%). However, there were no significant differences between RMD groups regarding symptomatic disease.

As shown in Table 2, most patients had mild disease, with fever as the most prevalent symptom. No patients had critical disease. Six patients required hospital admission (7.79% of all patients), with a median of 5 days duration (IQR 2–20) and one patient required pediatric intensive care. In each RMD diagnostic category there were two cases of hospital admission. Half of the admitted patients were on bDMARDS (2 on Infliximab, 1 on Canakinumab) (the sociodemographic and clinical characteristics of these patients are described in Table 3).

**Table 2:** Characteristics of patients with symptomatic COVID-19 symptoms

Variable	RMD Covid-19 patients *n* = 47
Women, n (%)	31 (65.95)
Age, mean (SD), years	12.04 (4.22)
Activity of the RMD, n (%)	
Mild	41 (87.23)
Moderate	5 (10.63)
Severe	1 (2.12)
COVID-19 symptoms, n (%)	
Dyspnea	2 (4.25)
Fatigue	10 (21.27)
Chest pain	5 (10.63)
Cough	17 (36.17)
Abdominal pain	2 (4.25)
Diarrhoea/vomiting	10 (21.27)
Fever	45 (95.74)
Anosmia	10 (21.27)
Dysgeusia	12 (25.53)
Joint pain	8 (17.02)
Headache	20 (42.55)
Odynophagia	16 (34.04)
Hospital admission required, n (%)	6 (12.76)

*Abbreviations*: *SD* standard deviation, *RMD* rheumatic and musculoskeletal diseases

**Table 3:** Characteristics of admitted patients with COVID-19

Variable	COVID-19 admitted patients *n* = 6
Women, n (%)	5 (83.33)
Age, median (IQR), years	14.5 (4–16)
Diagnosis, n (%)	
Systemic JIA	1 (16.6)
Oligoarticular JIA with associated uveitis	2 (28.8)
Systemic lupus erythematosus	1 (16.6)
Vasculitis	1 (16.6)
Autoinflammatory syndrome	1 (16.6)
COVID-19–related treatments during admission, n (%)	
Hydroxychloroquine	1 (16.6)
Glucocorticoids	2 (28.8)
Azithromycin	1 (16.6)
Lopinavir/ritonavir	1 (16.6)
Admission duration, median (IQR), (days)	5 (2–15)

*Abbreviations*: *IQR* interquartile range, *JIA* Juvenile idiopathic arthritis

The only patient who required admission to a Pediatric Intensive Care Unit had a recent diagnosis of systemic JIA, was on treatment with Canakinumab and glucocorticoids (> 10 mg prednisone/day), and had associated comorbidities. This patient developed pneumonia and a pleural effusion and was treated with antibiotic therapy and lopinavir/ritonavir. A catheter-associated right iliac vein appeared as a probably multifactorial complication. A few days later The patient’s condition improved after a few days and the recovery was complete at discharge.

No laboratory or imaging tests were performed for asymptomatic and mildly symptomatic patients and none received specific treatment for COVID-19.

### Multivariable model for symptomatic infection

The multivariable logistic regression model for symptomatic infection was adjusted for age, sex and previous comorbidities. None of the variables included in the final model were associated with a statistically significant result. Comorbidities dropped from the final model because predict failure perfectly. Exposure to DMARDs [biological (OR: 1.02 [0.34–3.09]; *p* = 0.95) or synthetic (OR: 1.24 [0.44–3.46]; *p* = 0.67)]), and exposure to glucocorticoids (OR: 1.27 [0.47–3.47]; *p* = 0.62)), did not achieve statistical significance and was not analysed in the final model. Although, long-term treatment with antimalarials seemed to be protective, significance was not demonstrated in the final model (OR: 0.11 [0.007–1.88]; *p* = 0.13). (Table 4).

**Table 4:** Multivariable logistic regression models. Associated factors for symptomatic infection and hospital admission

	Symptomatic infection Odd ratio (95% CI)	Hospital Admission Odd ratio (95% CI)
Age	1.04 (0.91 to 1.20)	1.06 (0.78 to 1.46)
Sex	2.03 (0.65 to 6.34)	0.38 (0.03 to 4.15)
Diagnosis: Multisystemic autoimmune disorders vs chronic inflammatory arthritis	1.55 (0.38 to 6.27)	2.09 (0.19 to 22.01)
Diagnosis: Autoinflammatory diseases vs chronic inflammatory arthritis	1.42 (0.33 to 6.09)	3.30 (0.16 to 66.15)
Glucocorticoids^a^	1.27 (0.47 to 3.47)	3.51 (1.04 to 11.84)*

^a^ prior COVID-19 exposure

* *p* < 0.05

### Multivariable model for hospital admission

In the multivariable logistic regression model adjusted for hospital admission, exposure to higher doses of glucocorticoids was the only variable associated with a higher odds of hospital admission. Exposure to DMARDs, −regardless of whether they were biological (OR: 1.97 [0.23–16.57]; *p* = 0.53) or synthetic (OR: 1.20 [0.15–9.56]; *p* = 0.86)-, did not reach statistical significance in the final model. Comorbidities were not analysed in the final model (OR: 3.45 [0.41–28.48]; *p* = 0.25). (Table 4).

## Discussion

This study conducted at six tertiary care referral hospitals presents the baseline characteristics and outcomes of pediatric patients with rheumatic diseases infected with SARS-CoV-2, contributing to the current knowledge in this area.

In the COVID-SORCOM registry, the majority (61%) of pediatric patients with RMD and COVID-19 had symptomatic infections. Fever, headache and cough were the predominant clinical features at presentation, with similar rates to those observed in a recent international, multicenter study of pediatric COVID-19 [18].

Presence of comorbidities was associated with an increased likelihood of symptomatic COVID-19 in patients with RMD, although under 8% of the patients required hospital admission.

Data from this study also reflect the uncertainties regarding treatment options for COVID-19 with different protocols emerging as knowledge of the disease progressed. In general, admitted patients received few experimental treatments for COVID-19 and in almost all cases symptoms resolved without serious or chronic complications. Patients exposed to disease-modifying agents did not appear to be at higher risk of severe disease or hospital admission due to COVID-19. However, results suggest that pediatric patients with RMD and chronic prior exposure to glucocorticoids have a greater risk of hospital admission, regardless of other factors. Although the number of patients in this study was limited, results are similar to data reported in adult patients with RMD and COVID-19 [6, 11]. Interpretation of data on glucocorticoid use may be challenging, as pediatric rheumatologists generally aim to maintain the lowest possible doses and patients with higher doses usually have more severe disease and/or higher disease activity.

The possibility of a less favorable outcome of COVID-19 in patients taking rituximab has recently been discussed [19–21]. In this cohort, only one patient with systemic lupus erythematosus had received rituximab in the 3 months prior to infection and, although presentation was with symptomatic disease, hospital admission was not required. This patient was also on hydroxychloroquine and mycophenolate mofetil, and the latter was discontinued until complete recovery from SARS-CoV-2 infection.

Although available data have shown that COVID-19 in children with RMD undergoing immunomodulatory treatment is not usually associated with severe disease, conventional clinical practice includes temporary interruption of immunomodulatory treatment when there is a concomitant infection [22]. In this cohort, most physicians maintained immunomodulatory treatment in patients with asymptomatic infections. Also, in most patients with symptomatic COVID-19, DMARDs (biological or synthetic) were maintained. Due to uncertainty, during the first wave the proportion of treatments which were discontinued was higher, but after this period, treatment was only stopped in very few cases. There is no evidence that continuing DMARDs during COVID-19 can alter the clinical course of SARS-CoV-2 infection and the benefits of keeping the disease inactive outweigh the risks [19, 22, 23].

The type of RMD seems to play an important role in the probability of hospital admission in adult patients and COVID-19, and patients with systemic autoimmune conditions seem to have the highest risk compared to patients with chronic inflammatory arthritis [6, 18]. However, this was not observed in the current pediatric population and a higher risk for any of the outcomes was not demonstrated.

No patients who required admission developed associated complications, except for an adolescent female with systemic lupus erythematosus and secondary antiphospholipid syndrome who had an episode of deep vein thrombosis of a lower limb. Thrombotic complications seem very uncommon in children with SARS-CoV-2, however, in adolescents with previous thrombotic risk factors thromboprophylaxis must be considered [24].

A large majority of children with asymptomatic disease were in remission. This reflects the importance of keeping the disease under control to avoid symptomatic COVID-19. Patients with high or moderate disease activity may be at greater risk for infections, including SARS-CoV-2.

The strengths of the study include the comprehensive analysis of pediatric patients with RMD and COVID-19 during the peak of the pandemic. All data were entered by the patients´ own rheumatology healthcare providers, the registry collects information on specific RMD diagnoses and includes cases from the major pediatric rheumatology units in Madrid suggesting that these findings may be more representative than single-centre studies..

However, the COVID-SORCOM registry is voluntary and does not capture all cases of COVID-19 in pediatric patients with RMD. This approach to data collection places limitations on causal conclusions and temporal relationships and therefore only limited inferences may be made based on these results. Due to the database design and inherent reporting bias the data may not be used to analyse the incidence of COVID-19 in this patient population.

Although the limited sample size makes this a descriptive study with exploratory analyses, detailed information on this population may be obtained. Preliminary results are in line with data published in the adult population with RMD, and may assist with the decision-making process and patient monitoring. Although further studies are required, children with RMD and/or receiving immunomodulatory therapies do not appear to have a significantly increased risk for severe COVID-19 and preventive measures should be similar to those recommended for the general population [22, 25].

In conclusion, overall outcomes in children and adolescents with RMD appear to be generally favorable, and most patients have non-severe infections. Rarely, COVID-19 may be severe in children and comorbidities and the use of glucocorticoids could be considered risk factors in the RMD pediatric population as well as in adults.

## Conclusions

Overall, no differences in COVID-19 outcomes in pediatric patients according to type of childhood-onset rheumatic disease were found. Some factors may increase the likelihood of complicated symptomatic infection and hospital admission. In this clinical registry, results were in concordance with data reported elsewhere in adult patients with RMD and COVID-19, and special caution should be exerted in patients with thrombotic risk, with comorbidities or with previous exposure to corticosteroids. This study provides novel results in childhood-onset rheumatic disease patients regarding susceptibility to moderate-severe COVID-19.

## Data Availability

The datasets generated and analyzed for the present study are available from the corresponding author on reasonable request.

## Abbreviations

ACE: Angiotensin-converting-enzyme inhibitors
CAPS: Cryopyrin-associated periodic syndrome
COVID-19: Coronavirus disease 2019
DMARD: Disease-modifying anti-rheumatic drug
csDMARDs: Classic DMARDs
ts/bDMARDs: Targeted synthetic or biologic DMARDs
FMF: Familial Mediterranean fever
JAK: Janus-Kinase
JIA: Juvenile idiopathic arthritis
HIDS: Hyperimmunoglobulin-D syndrome
IQR: Interquartile Range
MIS-C: Multisystem inflammatory syndrome in children
MKD: Mevalonate kinase deficiency
NSAID: Nonsteroidal anti-inflammatory drugs
PCR: Polymerase chain reaction
PFAPA: Periodic fever, adenopathy, pharyngitis and aphthous stomatitis
RMD: Rheumatic and musculoskeletal diseases
SARS: Severe acute respiratory syndrome
TNF: Tumor necrosis factor
TRAPS: TNF- receptor associated periodic syndrome
